# Risk factors for HIV infection in Males who have Sex with Males (MSM) in Bangladesh

**DOI:** 10.1186/1471-2458-7-153

**Published:** 2007-07-12

**Authors:** Philip A Chan, Omar A Khan

**Affiliations:** 1 Department of Internal Medicine, Brown University, Providence, Rhode Island 02912, USA; 2 Department of Family Medicine, University of Vermont, Burlington, Vermont 05401, USA

## Abstract

**Background:**

Recent surveillance data from Bangladesh indicate rising HIV infection among intravenous drug users (IDU) in the country. We suggest a likely association between HIV risk factors in this group and other groups, such as males who have sex with males (MSM).

**Methods:**

Data on MSM in Bangladesh was collected and analyzed from numerous primary and secondary sources, including government ministries, non-profit health organizations, and personal communications.

**Results:**

The overall prevalence of HIV in Bangladesh is relatively low, but surveillance data indicate that infection has reached significant proportions in certain high-risk groups and may soon spread to other groups, specifically MSM.

**Conclusion:**

The epidemiology of HIV infection in other countries suggests that increasing rates of HIV in higher-risk populations can precede an epidemic in the general population. We review the data concerning MSM, IDU and HIV in Bangladesh from a variety of sources and propose ways to prevent HIV transmission.

## Background

Bangladesh has a predominantly Muslim population of roughly 150 million people. Located between India and Burma, Bangladesh has a relatively low reported HIV prevalence in the general population, of less than 0.2% [1]. Due to the discrepancy between reported cases and actual cases, WHO/UNAIDS has estimated that approximately 7,500 people may actually have HIV in Bangladesh (Table 1). The first case of HIV was reported in a foreign drug smuggler in 1986 [2]. Since then, reported cases to the National AIDS Committee of Bangladesh have increased significantly to over 450 in 2003–2004 [3]. There are several groups at particularly high risk for HIV/AIDS in Bangladesh, and include intravenous drug users (IDU), commercial sex workers (CSW), males who have sex with males (MSM), truck drivers, migrant workers, and rickshaw pullers [3]. Within these groups, there is a disproportionate burden of disease, including sexually transmitted diseases (STDs). While the underlying social determinants of health such as poverty, access to medical care, education, and healthcare structure are most likely the root causes of these diseases, the more proximal causes include low condom use, high number of sexual partners, and relatively low HIV-related knowledge [4]. This represents a risk environment primed for the spread of HIV.

**Table 1:** Summary of HIV/AIDS in Bangladesh*

Total Population	149,664,000
Estimated number of people living with HIV/AIDS	7,500 (700 – 19,000)
Adult HIV prevalence	< 0.2%
At risk populations^‡^	2.0%
Reported HIV cases	465
Reported AIDS cases	87
Reported AIDS deaths	44

^‡^Populations include CSWs and people with STDs

*Data are from 2003–2004, WHO/UNAIDS

The prevalence of HIV in Bangladesh may be increasing substantially, specifically in IDUs [4]. National surveillance data for HIV from the Ministry of Health and Government of Bangladesh have reported data from a variety of groups showing the highest levels of HIV infection were identified among IDUs [5-8]. During the first four rounds of surveillance, from 1998 to 2002, the HIV prevalence increased from nearly zero to 4%. Reports from the recent fifth round of HIV surveillance demonstrate the prevalence of HIV in some groups of IDUs has risen to 8.9% [4].

Non-governmental studies have reported similar figures, including a prevalence of HIV of 5.6% among IDUs at a single treatment hospital in Tejgaon, Dhaka [9]. In contrast, the prevalence of HIV in CSWs has always been reported as being below 1%, according to national surveillance data [4]. Other survey data of brothels and CSWs have confirmed a low prevalence as well [10-12].

MSM are often overlooked as a high-risk population for HIV infection in Bangladesh. The role of MSM in the spread and transmission of HIV is not well studied and is limited by a number of factors: 1) Males may not report participating in sex with other males due to societal acceptance or identity issues; 2) Males may deny such behavior due to discrimination or stigma; 3) MSM may not report sexual behavior to health care workers. The structure of groups within MSM is not well characterized and the complexity of MSM interactions makes exact definitions a challenge. Indeed, a precise definition of MSM can be controversial, partly since the definitions and subgroups can vary by geographic region. To simply identify a male as "homosexual" overlooks many social and gender issues that may contribute to MSM behavior [13,14]. For instance, in the South Asian setting as elsewhere, the issue of sexual identity and sexual behavior do not always perfectly overlap [15]. Males that participate in MSM behavior on a regular basis may not consider themselves, or be considered by others, to be homosexual as they would in a more Western context. The terms *Kothi, Hijra*, and *Panthi *are basic physical descriptors of MSM in Bangladesh society, but encompass much more complex behavior and social relationships. Furthermore, the Western term "gay" may or may not be utilized as a self-descriptor among MSM and is especially true among MSM who engage in male-to-male sex but have an otherwise heterosexual identity in society. For the purpose of this paper, we will define MSM as males who have participated in anal intercourse with other males.

Three main groups are described within the context of MSM in Bangladesh and South Asia, and are known as *Kothis*, *Panthis*, and the *Hijra *[15,16]. The *Kothis *are usually males who are self-described as preferring the penetrated role in a coupling. Some in this group will sell sex to the *panthi *MSM who are the more masculinized males, preferring the penetrative/insertive or dominant role. *Kothis *are identified as a special group within their culture, and share both social and sexual relationships. The *Hijra *are a complex group, and encompass several physical descriptions including hermaphrodites, castrated males, and transgender individuals. This group has utilized the term *Hijra *as a self-descriptor across South Asia. *Hijra*s, more so than the other MSM groups, have a fairly rigid and defined social structure and do not usually associate with non-*Hijra *in a social setting. The notable exception to this is commercial sex work.

The *Kothis *and *Hijras *refer more to sexual identities within a larger group with a shared social networking versus the *Panthis*, which tends to describe sexual behavior more than sexual identity. *Panthis *are usually older males who may or may not be married, and who participate in sexual acts with other males. The relatively sparse reports on *Panthis *suggest that most participate in anal penetration (versus receptive) and do not necessarily self-identify as MSM. Regardless of the MSM group, low condom use, multiple partners, and a high rate of other STDs place these individuals at increased risk for HIV infection. Within this paper, data referring to "MSM" usually do not include *Hijra*; they include only *Kothi *and *Panthi*. Data for *Hijra *are presented separately, where available.

Studies from other South and Southeast Asian countries show that HIV epidemics start in locally isolated high-risk groups before spreading to the larger population. The neighboring countries of Bangladesh, specifically India and Myanmar (Burma), have seen HIV rates substantially increase in the last decade [17-19]. Studies have indicated dramatic rises in HIV, such as in CSWs in Bombay, India where the prevalence increased from 2% in 1984 to between 40–54% in 1992 [2,20]. In Manipur, India, HIV in IDUs increased from 0% to over 50% in a period of less than a year in 1989 [2,21,22]. However, these rises likely represent an improvement in detection, and small sample sizes, rather than accurate estimates of MSM risk. In Myanmar, the WHO reports a HIV seropositive rate (in 2004) of 27% and 30% among CSWs and IDUs, respectively [23]. The large migrant worker population and porous borders of Bangladesh are a significant risk factor for HIV transmission and may facilitate travel and infection between these countries.

Previous work in Bangladesh has identified HIV as a concern among IDUs in Bangladesh [5-7,9] and among MSM in India and Pakistan [24,25]. There has not been a clear link between the high risk groups of MSM and IDUs in Bangladesh, and this is the relationship we explore in this paper. We recognize the need for high-quality regional, national and sub-national surveillance among a variety of at-risk groups. We also acknowledge the barriers to this, and discuss these in a later section. Much of the data is gathered by government organizations or international health groups, and is not published in peer-reviewed literature. Further, prevalence figures across years can be limited by small or non-comparable sample sizes and inadequate calculation or reporting of confidence intervals. This presents a challenge to analysis and subsequent policymaking. From our work in this area, and access to the available data, we present what is currently accessible but suggest that care be taken when interpreting the information. We have extrapolated from the data when appropriate, for instance, in the provision of 95% confidence intervals, which were not available to us from the source data.

## Methods

Data on prevalence of HIV infection and other STDs was obtained from the surveillance data published by the Bangladesh Ministry of Health. This was collected by a variety of public health organizations in collaboration with the Bangladesh Ministry of Health. A total of six rounds of surveillance data have been conducted from locations across Bangladesh from 1998 to 2005. The first five rounds initially consisted of epidemiological and behavioral data, whereas the most recent round only gathered epidemiological data. The latter specifically looked at the prevalence of HIV and syphilis among several "high-risk" groups. Behavioral data was gathered by the National HIV Surveillance System of Bangladesh and consisted of responses to knowledge, attitude, behavior and practice (KABP) questions. Examples include respondents' knowledge of high-risk activity within these groups, such as how HIV may be transmitted, and the appropriate use of condoms during intercourse. High-risk groups studied include IDUs, heroin smokers, female sex workers (brothel, street, hotel, and casual sex workers), MSM (as addressed in the Background section, includes male sex workers and non-sex workers), *Hijras *(includes transgender), truckers, dock workers, and rickshaw pullers. The country was divided into six geographical regions for study purposes: Central, Northwest, Northeast, South, Southeast, and Southwest. Within each geographical region, different cities were selected as testing sites and referred to by a letter designation such as "Central A" or "Central B". The sample size was calculated at 380 subjects per site assuming an HIV prevalence rate of approximately 1% and a 95% confidence interval. At clinics with high attendance rates, the first four hundred individuals who came to the clinic were included for analysis. At sites with lower attendance, all available individuals were included. During the latest round in 2006, 11029 samples were collected from the pre-defined high-risk groups including IDUs (20 sites), CSWs (18 sites), MSWs (1 site), MSM (3 sites), *Hijras *(2 sites), and other groups (3 sites). HIV testing procedures and practices are outlined elsewhere [4].

A complete data review was conducted from literature searches of the databases MEDLINE and POPLINE, accessible online. Keywords used in the search included ''Bangladesh'', ''HIV'', ''AIDS'', ''MSM'' (and ''males who have sex with males''), ''IDU'' (and ''intravenous drug user''), ''CSW'' (and ''commercial sex workers''). Comparative information from regional neighbors such as Pakistan and India were obtained by entering those countries as search terms. All data available from 1986 (the year of the first reported HIV case in Bangladesh) to 2006 were reviewed.

Other data were obtained from personal communication with the Naz Foundation International (NFI), the major non-governmental organization working with MSM in South Asia, through communication with other international organizations including UNAIDS Bangladesh, and reports from the Bangladesh Ministry of Health

## Results

The available data from six rounds of national surveillance for HIV in Bangladesh represents the most complete information for high-risk groups since 1998 (Table 2) [4]. HIV prevalence among IDUs has been low in most geographical locations in Bangladesh (0% in Southwest, Northwest, and South during the first five rounds). The exception is the Central region, which has shown an increasing prevalence of HIV infection during the last five rounds of surveillance at 1.4% and 4.9% in 1999 and 2005, respectively (95% CI: 0.27 – 2.53 and 3.60 – 6.20, respectively). Other regions have reported HIV cases for the first time in the most recent round of surveillance (Northwest-F1, 2% [1 of 49, 95% CI: -1.92–6.00] and Southwest-D, 1% [1 of 159, 95% CI: -0.60–1.86]). Among certain areas in Central Bangladesh, the HIV prevalence reached as high as 8.9% (4 of 45, 95% CI: 0.57 – 17.21) [4].

**Table 2:** HIV prevalence in Bangladesh, selected surveillance regions

	**Round II**	**Round III**	**Round IV**	**Round V**	**Round VI**
**Year range**	1999–2000	2000–2001	2002	2003–2004	2004–2005
**Injection Drug Users, Central**	1.4% (6/418) (CI: 0.27 – 2.53)	1.7% (7/401) (CI: 0.46 – 2.98)	4.0% (16/403) (CI: 2.06 – 5.88)	4.0% (16/404) (CI: 2.06–5.86)	4.9% (52/1061) (CI: 3.60 – 6.20)
**Male Sex Workers, Central**	ND	0% (0/310)	0% (0/401)	0% (0/274)	0% (0/235)
**MSM, Central**	ND	0% (0/399)	0.2% (1/406) (CI: -0.24 – 0.74)	0% (0/399)	0% (0/405)
***Hijra*s, Central**	ND	ND	0.8% (3/393) (CI: -0.10 – 1.62)	0.2% (1/405) (CI: -0.24 – 0.74)	0.8% (3/381) (CI: -0.10 – 1.68)
Total*	0.2% (8/4338) (CI: 0.05 – 0.31)	0.2% (14/7063) (CI: 0.10 – 0.30)	0.3% (27/7877) (CI: 0.21 – 0.47)	0.3% (35/10445) (CI: -0.10 – 1.62)	0.6% (70/11,029) (CI: 0.48 – 0.78)

*Includes other high-risk populations (as defined by surveillance report) such as female CSWs, Truckers, Rickshaw pullers, Dock workers

**95% Confidence Intervals are provided

No HIV cases have been reported among individual groups of MSM or male sex workers (MSW) (Table 2). In undifferentiated groups, one case of HIV in each of two centers was reported out of 283 and 231 people in Southeast and Northeast Bangladesh, respectively. Among the *Hijra*, the last three rounds of surveillance have reported HIV infection rates of 0.8% (3/393, 95% CI: -0.10 – 1.62), 0.2% (1/405, 95% CI: -0.24 – 0.74), and 0.8% (3/381, 95% CI: -0.10 – 1.68), respectively.

The rate of syphilis among MSM in Central Bangladesh during the last four rounds has been 1.8% (7/399, 95% CI: 0.46 – 3.04), 0.7% (3/406, 95% CI: -0.09 – 1.57), 1.5% (6/399, 95% CI: 0.31 -2.69), and 2.0% (8/405, 95% CI: 0.62 – 3.34), respectively. For MSW, the rate of syphilis has ranged from 7.7% (24/310, round III, 95% CI: 4.77 – 10.71), 3.2% (13/401, round IV, 95% CI: 1.51 – 4.97), 6.2% (17/274, round V, 95% CI: 3.34 – 9.06), and 3.8% (9/235, round VI, 95% CI: 1.38 – 6.28). Syphilis rates in the *Hijra *community have been 10.4% (41/393, 95% CI: 7.41 – 13.45), 10.4% (42/405, 95% CI: 7.40 – 13.34) and 5.2% (20/381, 95% CI: 3.01 – 7.49) for the last three rounds, respectively [4].

There is significant interaction observed between IDUs, CSWs, and MSM (Table 3). The range of responses across all geographical locations in Bangladesh during the fourth round of HIV/AIDS surveillance indicate that between 18–54% (95% CI: 15.63 – 20.37, 50.92 – 57.08) of IDUs bought sex from a CSW in the past week while only 17–31% (95% CI: 14.68 – 19.32, 28.14 – 33.86) of those IDUs used a condom at their last encounter. Only 8–15% (95% CI: 6.33 – 9.67, 12.80 – 17.20) consistently used condoms. Ten percent (95% CI: 8.15 – 11.85) of male IDUs had sex with another male, and only 12% (95% CI: 9.99 – 14.01) of these IDU reported a condom being used by either partner during the encounter [4]. Further information based on the type of sexual encounter was not available.

**Table 3:** IDUs: Relationship to CSWs and MSM

Used injection drugs in past year	100%
HIV infection	0–4% (CI: 0, 2.79 – 5.21)
Sold sex in past week	2–9% (CI: 1.14 – 2.86, 7.23 – 10.77)
Bought sex from a sex worker in past week	18–54% (CI: 15.63 – 20.37, 50.92 – 57.08)
Condom use at last sex act with CSW	17–31% (CI: 14.68 – 19.32, 28.14 – 33.86)
Consistent condom use during last year	8–15% (CI: 6.33 – 9.67, 12.80 – 17.20)
Male IDU who had sex with MSM	10% (CI: 8.15 – 11.85)
Used condom with MSM	12% (CI: 9.99 – 14.01)

*National AIDS/STD Programme, 2004–2005, page 8; Fourth round of HIV surveillance

Data pooled from three groups of IDU: Central (n = 403), Northwest-A (n = 405), and Northwest-B (n = 200)

** 95% Confidence Intervals are provided

Based on government surveillance data from 2001, the rate of MSM always using condoms is 9% (Central, 33/366, 95% CI: 6.07 – 11.93), 1% (Southeast, 3/328, 95% CI: -0.08 – 2.08), and 51% (*Hijra*, Central, 197/387, 95% CI: 46.02 – 55.98), respectively (Table 4). The percent of MSM who did not use any protective method was 57% (Central, 209/366, 95% CI: 51.93 – 62.07), 18% (Southeast, 59/328, 95% CI: 13.84 – 22.16), and 15% (*Hijra*, Central, 58/387, 95% CI: 11.44 – 18.56) [4]. A separate survey conducted by the Population Research and Development Associates (PRDA) of Bangladesh reported that 58% (94/162, 95% CI: 50.42 – 65.62) of MSM reported use of condoms during sex. However, only 4.3% (7/162, 95% CI: 1.18 – 7.42) of respondents had regular blood tests for HIV [26]. In another survey conducted by the Naz Foundation International of 200 MSM at a drop-in center in Northeast Bangladesh, only 33% (66/200, 95% CI: 25.48 – 39.52) and 31% (62/200, 95% CI: 24.59 – 37.41) of males used condoms in insertive and receptive acts, respectively. Furthermore, 78% (156/200, 95% CI: 72.26 – 83.74) of males had greater than ten partners in the last month and 21% (42/200, 95% CI: 15.35 – 26.65) of males had 51+ partners in the last month [16].

**Table 4:** Males using condoms during commercial sex with other males*

	**MSM (Central)**	**MSM (Southeast)**	***Hijra *(Central)**
	n = 366	n = 328	n = 387

**Did nothing to protect**	57% (209/366) (CI: 51.93 – 62.07)	18% (59/328) (CI: 13.84 – 22.16)	15% (58/387) (CI: 11.44 – 18.56)
**Washed in urine or Dettol (an antiseptic)**	12% (44/366) (CI: 8.67 – 15.33)	37% (121/328) (CI: 31.77 – 42.23)	50% (194/387) (CI: 45.02 – 54.98)
**Always used a condom**	9% (33/366) (CI: 6.07 – 11.93)	1% (3/328) (CI: -0.08 – 2.08)	51% (197/387) (CI: 46.02 – 55.98)

*95% Confidence Intervals are provided

The association between MSM and non-male partners is also high (Table 5). Government surveillance data for MSM in Central and Northeast Bangladesh indicate the mean number of female commercial sex partners was five and four, respectively. In Central and Northeast Bangladesh, 91% (544/598, 95% CI: 88.71 – 93.29) and 61% (270/442, 95% CI: 56.45 – 65.55) bought sex in the last month, respectively. Thirty-four percent (Central, 203/598, 95% CI: 30.20 – 37.80) and 43% (Northeast, 190/442, 95% CI: 38.38 – 47.62) of MSM had sex with a female commercial partner, while 62% (Central, 371/598, 95% CI: 58.11 – 65.89) and 18% (Northeast, 80/442, 95% CI: 14.42 – 21.58) had sex with a non-commercial female partner in the last month. There was also a high rate of group sex at 36% (Central, 215/598, 95% CI: 32.15 – 39.85) and 25% (Northeast, 111/442, 95% CI: 20.96 – 29.04) [4].

**Table 5:** Selected indicators pertaining to sexual behavior of MSM in Bangladesh*

	**MSM (Central)**	**MSM (Northeast)**
	n = 598	n = 442

**Sold sex last week**	0%	42% (186/442) (CI: 37.40 – 46.60)
**Bought sex last week**	78% (466/598) (CI: 74.68 – 81.32)	57% (252/442) (CI: 52.38 – 61.62)
**Bought sex last month**	91% (544/598) (CI: 88.71 – 93.29)	61% (270/442) (CI: 56.45 – 65.55)
**Sex with female commercial partner last month**	34% (203/598) (CI: 30.20 – 37.80)	43% (190/442) (CI: 38.38 – 47.62)
**Sex with female non-commercial partner last month, including wife**	62% (371/598) (CI: 58.11 – 65.89)	18% (80/442) (CI: 14.42 – 21.58)
**Sex with female non-commercial partner last month, excluding wife**	45% (269/598) (CI: 41.01 – 48.99)	11% (49/442) (CI: 8.08 – 13.92)
**Sex with male and female partners last year**	78% (466/598) (CI: 74.68 – 81.32)	48% (212/442) (CI: 43.34 – 52.66)
**Group sex last month**	36% (215/598) (CI: 32.15 – 39.85)	25% (111/442) (CI: 20.96 – 29.04)

*Third round of HIV surveillance, Bangladesh. Confidence intervals are for 95% confidence.

The knowledge of MSM about the transmission of HIV/AIDS varies by location and group. Seventy-nine percent of MSM in Central Bangladesh (472/598, 95% CI: 75.74 – 82.26) in the government surveillance survey could name at least two correct modes of HIV transmission (male to female sex, male to male sex, needle sharing, mother-to-child birth, mother-to-child breastfeeding, sex without condoms, or blood transfusions), while 46% of MSM in Northeast Bangladesh (203/442, 95% CI 41.35 – 50.65) and 33% of *Hijra *in Central Bangladesh (125/380, 95% CI: 28.27 – 37.73) could do the same [4]. In the Naz Foundation International Survey of 200 MSM in Northeast Bangladesh, 31% (62/200, 95% CI: 24.59 – 37.41) had not heard of HIV/AIDS and 81% (162/200, 95% CI: 75.56 – 86.44) of MSM were unaware of their own personal risk factors for infection [16].

## Discussion

There are a number of factors that place susceptible groups in Bangladesh at high risk for a rapid increase in HIV infection, and by extension, lead to an expanded epidemic of HIV in the general population and other at-risk groups. Among the social factors underlying HIV, both social stigma and poverty affect the high-risk groups we discuss in this paper. Not only is stigma present once the diagnosis of HIV/AIDS has been made, it also applies in the Bangladesh setting to the generally hidden behavior of male-to-male sex, which becomes a risk factor for contracting the disease when unsafe. The geography of Bangladesh also contributes to the transmission of HIV in a number of ways. One is the obvious proximity of Bangladesh to neighboring countries with high HIV rates and injection drug use. Secondly, Bangladeshi migrant laborers compose a separate risk group. Away from their homes for extended periods of time, these workers have been recognized as an at-risk population for contracting HIV through unsafe sexual practices while abroad, including commercial sex with either males or females, and unknowingly infecting their (usually female) partners at home [27]. One of the main biomedical factors affecting the risk profile of Bangladesh is unequal and unavailable access to healthcare, which includes HIV antiretroviral therapy. Treatment for HIV is generally not available in Bangladesh, although it can be acquired by individuals of means, at a high price (UNAIDS, personal communication).

HIV has been detected in most of the surveyed regions of Bangladesh, according to the results reported above. There are important limitations to these regional data. While the surveys have been increasingly comprehensive, the sample sizes may preclude valid subgroup analysis. The sample sizes also mean that regional comparisons, especially when dealing with subgroups of less than a hundred individuals, are less valid than regional prevalence as a whole. Indeed, perhaps the clearest generalization one can make is that HIV infection among IDUs in Bangladesh has been increasing since the government-led surveillance was initiated, and it is likely that the Central region is currently the most susceptible [4].

In the most recent round of surveillance, HIV has also been measured in IDU groups for the first time in other regions of Bangladesh (Northwest and Southwest regions). Given the inherent high risk of IDUs contracting HIV, it is plausible that increased HIV rates would be seen in this group as a sentinel alert, prior to detectable spread in the general population. We suggest this progression is inevitable when: (1) there is a relatively low understanding of high-risk behaviors in the general population, as borne out by the behavioral data; (2) there is inter-mixing of high-risk groups and the general population through conduits such as unprotected commercial sex, as suggested by studies of both migrant workers and of the interactions between MSM and female CSW.

The increasing prevalence of HIV among IDUs may predict a more general increase of HIV in the Bangladesh population over the next several years. Although the current rate of HIV infection in MSM remains low in absolute numbers among both street and brothel workers, the increasing rate of HIV in IDU, and the proportion of reported IDUs (10%) who participate in sex with MSM, suggests a possible means for the virus to spread across these vulnerable groups.

There are several limitations to this study. Much of the data gathered comes from groups of people that are assumed to represent the larger populations. Many of the tested groups are of small sample sizes and report no HIV, which is likely an underestimation of the true prevalence of disease. Furthermore, the data reported from the government organizations do not always include statistical calculations of variance and standard deviation. In addition, while this is no means an issue unique to Bangladesh, it should be noted that the path to publication of the surveillance data is not always a straightforward one. The data may be published in internal report form, selected data may be presented at international meetings, and subsets of the data may be controlled by the scientific and public health partners who collaborated on the overall study. There is an opportunity here, and a caution. The opportunity is for diverse groups to collaborate not just on study design, data collection, and analysis, but also to participate in sharing this information with the broader public health community. The caution is that if data is sequestered, or made incompletely available, it may a) lose its timeliness when it is finally made available, and b) contribute to mistrust between the at-risk populations and the public health bodies which purport to protect them.

As we have mentioned, the data must be interpreted with caution; however, the available data are highly suggestive of increasing HIV prevalence in Bangladesh as a whole and within these high-risk groups. They also indicate that populations of MSM and IDU warrant particular interventions to minimize risk for HIV.

Other factors contribute to the difficulty in gathering accurate quantitative and qualitative data from these groups. High-risk populations are historically difficult to analyze due to isolation from mainstream society. This problem is compounded by cultural and religious factors that are unfamiliar to many who traditionally operate within a Western construct of public health. For example, little is known about the *Panthi *group of MSM; due to their preference for the insertive role, they are more likely to be behaviorally bisexual, but the elusive nature of this group and others makes accurate socio-behavioral depiction of HIV challenging. However, we suggest that some of these characteristics can also present an opportunity for prevention, e.g., the social networking of MSM groups as seen among *Hijras *may make HIV prevention strategies easier to implement, provided adequate trust is built up and the aims of the health workers and the at-risk individuals are closely aligned. This strategy has been utilized by NGO work in the region, in particular that of the Naz Foundation and its partner agencies.

Other strategies that may be useful in preventing HIV transmission include targeting specific high-risk groups versus the general population due to the current low rates of HIV infection in Bangladesh. Such approach would be more practical and beneficial than a larger and more general approach. High risk groups including IDUs, CSWs, and MSM are the most likely to experience HIV epidemics initially, and small groups of IDUs are already beginning to show disturbing signs of this trend.

The PRDA survey discussed in the Results section (conducted from 2003 to 2004) also found a significant number, 399 in all, of non-government organizations (NGOs) participating in HIV prevention activities throughout all 64 districts of Bangladesh. Of these, 134 were local, 225 were national, and 40 were international [26]. While many provided health awareness programs (89%), only 11% (44/399) provided voluntary counseling and testing (VCT), and 7% (29/399) actually sent blood for HIV testing. Furthermore, only 9% of NGOs reported receiving IDU patients and only 1% housed needle exchange programs.

Several NGO-based programs in Bangladesh specifically exist to help prevent the spread of HIV in MSM. The Bandhu Social Welfare Society, a partner of the Naz Foundation International, addresses the needs and risks of MSM via a community-based and peer-led approach. This group also conducts outreach and prevention programs in a number of locations throughout the country. However, their success is limited to the relatively small population they serve, and their work has recently been curtailed by a paucity of continued donor funding.

The range of practical interventions should follow a logical continuum of systems-based policies affecting individual-based behavior through community-based implementation. As is practiced by the better programs, education and empowerment needs to be the foundation for prevention efforts to decrease the spread of HIV among high-risk groups. Targeted intervention of specific groups such as IDUs and MSM should be the focus of current resources, and needs to include VCT and distribution of needles and condoms, which are only being done by a few NGOs at present. While recognizing economic barriers to antiretroviral provision in Bangladesh, it would be irresponsible not to call for greater and more equitable access to these lifesaving therapies which are readily available in the West.

## Conclusion

The available data from Bangladesh indicate the presence of HIV among MSM and Hijras, and are suggestive of increasing HIV prevalence among the high-risk groups of IDUs and CSWs. MSM in Bangladesh are at increased risk for HIV infection due to sexual behavior including low condom use and association with IDUs. Trends from other settings suggest that that HIV will spread among these high-risk groups before spreading to the general population. In reviewing the current data in this area, we propose there is still the time and opportunity for increased cooperation among the health, population and development sectors to develop targeted prevention strategies and ethical treatment modalities for these groups.

## Competing interests

The author(s) declare that they have no competing interests.

## Authors' contributions

PC and OK both contributed equally to this work. Both authors have read and approved the final manuscript.

## Pre-publication history

The pre-publication history for this paper can be accessed here:


